# Effects of high-intensity interval training and moderate-intensity continuous training on classroom attention in fifth-grade students

**DOI:** 10.7717/peerj.21580

**Published:** 2026-07-21

**Authors:** Zhao Zhang, Wenhua You, Dexin Wang, Yuankun Long, Yu Zhe

**Affiliations:** 1 Physical Education Institute, Xi’an University of Architecture and Technology, Xi’an, Shaanxi, China; 2 College of Competitive Sports, Shanghai University of Sport, Shanghai, China; 3 College of Physical Education, Dalian University, Dalian, Liaoning, China

**Keywords:** High-intensity intermittent, Continuous aerobic exercise, Grade Five, Attention

## Abstract

**Objective:**

The aim was to examine how high-intensity interval training (HIIT) and moderate-intensity continuous training (MICT) differentially affect in-class attention among fifth-grade students, with the goal of informing evidence-based physical education curriculum design.

**Methods:**

Seventy-two children aged 10–11 years were randomly assigned to three groups: HIIT, MICT, and a control group (*n* = 24 each). The HIIT group completed a 6-week intervention with three 24-minute sessions per week (≥85% maximum heart rate (HRmax)), while the MICT group performed continuous running at 60–69% HRmax at the same frequency. The control group maintained regular school activities. Exercise intensity was monitored using Polar heart rate sensors. Attention was assessed pre- and post-intervention via an intelligent video-based system analyzing four sub-indicators: head posture, gaze direction, eye openness, and facial detection, along with an overall attention score. Data were analyzed using repeated-measures analysis of variance (ANOVA).

**Results:**

Both HIIT and MICT significantly improved the overall attention score (*P* < 0.01). The HIIT group increased from 0.39 ± 0.08 to 0.70 ± 0.14 (a 79.5% improvement), while the MICT group rose from 0.38 ± 0.10 to 0.67 ± 0.12 (a 76.3% improvement). The HIIT group demonstrated a significantly greater improvement than the MICT group (*P* < 0.01). The control group showed minor gains (31.0%; *P* < 0.05). Sub-indicator analysis revealed that HIIT significantly enhanced head posture and gaze direction (*P* < 0.01), while MICT primarily improved facial detection (*P* < 0.01).

**Conclusion:**

Both high-intensity interval training (HIIT) and moderate-intensity continuous training (MICT) demonstrated positive effects on the enhancement of children’s overall in-class attention. Notably, HIIT demonstrated superior overall effects and produced significantly greater improvements across multiple attention sub-dimensions compared with MICT. These findings provide empirical support for the incorporation of HIIT into school-based physical education curricula as an effective strategy for enhancing students’ cognitive performance.

## Introduction

Attention is the ability of an individual’s mental activities to direct and focus on specific things (Petersen & Posner, 2012). It is an important component of cognitive function, and its development has a profound impact on the improvement of children’s learning ability and their physical and mental health growth (Gallen et al., 2023). Research indicates that attention is composed of four dimensions: attention span, stability, allocation capacity, and shifting efficiency (Donnelly et al., 2016). In terms of its types, it can be further classified into selective, persistent, spatial and divisive attention (Funayama, 2024). Its developmental level is directly linked to the maturity of the nervous system and shows a significant positive correlation with classroom attention performance (Song et al., 2023). During the learning process, the efficient operation of attention can promote the efficiency of information processing (Zhu et al., 2023). Neuroscience research indicates that the primary and secondary school stage (7 to 15 years old) is a crucial window period for the development of attention (Liang et al., 2022), during which children’s nervous systems have a high degree of plasticity. Recent evidence indicates that structured exercise interventions can enhance attention and executive function in children. These improvements are thought to arise from exercise-induced increases in neurotransmitter availability and from enhanced activation and functional connectivity in prefrontal and related neural networks (Liu et al., 2025).

Physical activities of children and adolescents during school have a multi-dimensional promoting effect on the development of classroom attention (Janssen et al., 2014). In school settings, opportunities for physical activity are distributed across multiple environments, including physical education (PE) lessons, recess, and playground activities. It should be noted that PE classes primarily follow national curricular objectives and are not designed solely to increase daily physical activity levels (Webster, 2023). The World Health Organization recommends that children aged 5 to 17 engage in at least 60 min of moderate to vigorous physical activity every day (Ma, Le Mare & Gurd, 2015). Existing research indicates that systematic intervention in school physical education courses can significantly improve students’ attention performance in the classroom, especially in physical education courses with moderate complexity and intensity (Heemskerk et al., 2020). Relevant studies have confirmed that systematic intervention in physical education courses can significantly improve students’ performance in classroom task behaviors. Among them, the continuous aerobic exercise program combining high complexity and moderate to high intensity has a more significant effect on enhancing attention (Granacher & Borde, 2017). Meanwhile, although recess and playgrounds provide natural opportunities for movement, previous studies have identified substantial barriers, with many children failing to achieve recommended activity intensities during school hours (Zhang et al., 2025). In the present study, the term “classroom” refers specifically to the setting in which attention outcomes were assessed, rather than the location of exercise implementation. These contextual constraints underscore the need for structured and time-efficient exercise strategies that can be feasibly integrated into existing school schedules.

In order to compared the effects of high-intensity interval training (HIIT) and moderate-intensity continuous training (MICT) on health and cognitive outcomes in children and adolescents. A recent systematic review and meta-analysis involving 26 intervention studies (1,078 participants aged 9–19) showed that both HIIT and MICT significantly improved multiple cardiometabolic outcomes in overweight and obese youth, but HIIT was more effective in enhancing cardiorespiratory fitness (VO_2_max) and reducing systolic blood pressure compared with MICT (Zheng et al., 2025). Similar evidence from earlier meta-analytic work supports that HIIT induces greater improvements in cardiorespiratory fitness than MICT across a broader pediatric age range, with a pooled effect size favoring HIIT (SMD = 0.51) (Cao, Quan, Zhuang, 2019). Beyond cardiometabolic outcomes, individual randomized controlled trials have also examined physiological and cognitive outcomes. For example, short-term HIIT has been shown to produce superior improvements in cardiac autonomic function compared to MICT in healthy children aged 10–13 years (Van Biljon et al., 2018), indicating that HIIT may more profoundly enhance autonomic regulation of the heart. In the domain of cognition and behavior, exercise interventions comparing HIIT and MICT in children with attention-deficit/hyperactivity disorder attention-deficit/hyperactivity disorder (ADHD) have reported that both training types significantly improve behavioral inhibition and reaction time, but HIIT demonstrates additional benefits for reducing attention deficits and enhancing cognitive task performance relative to MICT (Sabaghi et al., 2025).

Therefore, HIIT and MICT should be integrated into the teaching organization form of physical education classes, not only aiming to improve physical fitness, but also to promote the development of motor ability, cognitive participation and self-regulation skills through structured motor tasks. After being incorporated into physical education classes, HIIT and MICT not only differ in terms of exercise intensity, but also in the organization of classroom activities, time structure, and the cognitive needs of students. Recent studies have shown that physically active interventions with high cognitive engagement can be effectively integrated into youth exercise and educational settings, and may produce beneficial effects on executive functions, particularly inhibitory control and working memory (Mao et al., 2024). These findings suggest that the cognitive benefits of HIIT may depend not only on physiological intensity but also on how the exercise tasks are designed and sequenced in the course context.

In this context, this study aims to compare the effects of implementing a 6-week HIIT and MICT intervention in regular physical education classes on the overall attention and its sub-components of fifth-grade students.

By integrating cognitive assessment with physiological control, this study not only explores the differentiated impact of exercise intensity on attention performance, but also provides empirical evidence for curriculum design and teaching implementation on how to scientifically integrate high-intensity intervals and moderate-intensity continuous training into school physical education courses.

## Participants & Methods

### Study design

This study adopted a randomized controlled design to examine the effects of different exercise intensities on in-class attention in fifth-grade children. The intervention was conducted within regular school physical education settings over a 6-week period. Participants were randomly assigned to a high-intensity interval training group (HIIT), a moderate-intensity continuous training group (MICT), or a control group, and measurements were obtained before and after the intervention.

### Participants

A total of 72 fifth-grade students (aged 10–11 years) were recruited from a primary school. Sample size estimation was performed using G*Power 3.1 based on a repeated-measures ANOVA (within–between interaction). In the absence of pilot data, a medium effect size (*f* = 0.25) was selected based on previous school-based exercise intervention studies reporting small-to-moderate effects on cognitive outcomes in children (De Greeff et al., 2018). The significance level was set at *α* = 0.05 and statistical power at 0.95, with three groups and two time points, yielding a minimum required sample size of 66. To account for potential attrition, 72 participants were ultimately included.

Inclusion criteria were:

(1) Age between 10 and 11 years;
(2) Body mass index (BMI) within the normal range for age and sex;
(3) No history of cardiovascular, respiratory, neurological, or musculoskeletal disorders;
(4) Absence of diagnosed neurodevelopmental or psychological conditions;
(5) No participation in structured extracurricular physical training during the study period. Participants missing three or more intervention sessions were excluded from analysis.

BMI was calculated as body weight (kg) divided by height squared (m^2^). Normal BMI was defined as the 5th-85th percentile for age and sex according to Chinese national reference standards.

This study was approved by the Scientific Research Ethics Committee of Shanghai University of Sport (Approval No. 10Z772023RT094). Written informed consent was obtained from all participants and their guardians.

### Methods

### Exercise intervention methods

This study was conducted between October and December 2024 with a cohort of 72 fifth-grade students (aged 10–11 years), recruited from a single elementary school. Participants were stratified by sex and randomly assigned in a 1:1:1 ratio to one of three parallel groups: (1) the high-intensity interval training group (HIIT; *n* = 24, 12 boys and 12 girls), (2) the moderate-intensity continuous training group (MICT; *n* = 24, 12 boys and 12 girls), or (3) the control group (CON; *n* = 24, 12 boys and 12 girls). Written informed consent was obtained from all legal guardians prior to enrollment, and assent was obtained from each participating child. Baseline assessments (Table 1) confirmed no statistically significant intergroup differences across nine key variables: sex distribution, age, body mass index (BMI), vital capacity, 50-m sprint time, sit-and-reach distance, 1-min rope skipping count, 1-min sit-up count, and 50-m ×8 shuttle run time (all *p* > 0.05), indicating successful baseline homogeneity. Furthermore, all participants demonstrated age-appropriate cognitive functioning, as verified through standardized teacher observations and classroom performance records.

**Table 1:** Homogeneity test results of statistical variables and physical and mental health status in fifth-grade children (M ± SD).

Categories	Total (*n* = 72)	Control (*n* = 24)	High-intensity interval training (*n* = 24)	Moderate-intensity continuous training (*n* = 24)	ANOVA	
					*F*	*p*
Gender (Male/Female)	72 (36/36)	24 (12/12)	24 (12/12)	24 (12/12)	∼	∼
Age	11.53 ± 0.53	11.46 ± 0.51	11.46 ± 0.51	11.67 ± 0.56	1.245	0.294
Intelligence	Normal	Normal	Normal	Normal	∼	∼
BMI (Kg/m^2^)	18.38 ± 2.01	17.72 ± 2.62	18.5 ± 1.45	18.93 ± 1.64	2.355	0.103
Vital capacity (ml)	1,992.92 ± 508.46	1,839.58 ± 597.64	2,087.5 ± 357.91	2,051.67 ± 524.72	1.7	0.19
50 m Sprint (s)	9.3 ± 0.65	9.12 ± 0.66	9.44 ± 0.57	9.33 ± 0.68	1.559	0.218
Sit-and-Reach (cm)	8.46 ± 1.49	8.26 ± 1.33	8.82 ± 1.7	8.31 ± 1.41	1.054	0.354
1 min Rope skipping (counts)	95.78 ± 16.35	90.71 ± 20.72	100.67 ± 13.53	95.96 ± 12.67	2.312	0.107
1 min Sit-ups (counts)	34.9 ± 5.21	33.83 ± 4.77	35.13 ± 5.12	35.75 ± 5.73	0.841	0.435
50 m × 8 Shuttle Run (s)	115.22 ± 10.21	116.88 ± 10.23	117.54 ± 9.33	111.25 ± 10.24	2.9	0.062

**Intervention schedule:** The study implemented an 8-week exercise intervention program from October to December 2024, executed in three phases: week 1 will complete baseline testing (pre-intervention attention tests, physical fitness assessment) and a pilot study (eight children randomly selected from a non-intervention group, half boys and half girls, to verify the training protocol’s physiological adaptability and movement feasibility); weeks 2–7 will implement the 6-week exercise intervention; the intervention was conducted three sessions per week on non-consecutive days, for a total of 18 sessions. Week 8 will conduct post-intervention attention assessments. A dynamic venue adjustment mechanism will be used: the outdoor playground will be utilized during sunny weather, switching to the indoor gymnasium during rainy conditions.

**Exercises intervention design:** Regarding the MICT group protocol design, existing studies have demonstrated that aerobic running can improve children’s attention, with moderate-intensity interventions showing the best effects (Li et al., 2025). For the HIIT group protocol design, movements such as jumping jacks, cross jumps, vertical jumps, and burpees have also been proven to positively impact children’s attention (Ma, Le Mare & Gurd, 2015). Simultaneously, referencing studies by Martínez-López et al., (2018) and Mezcua-Hidalgo et al. (2019), the work-to-rest ratio was set at 1:1 (*i.e.,* 30 s:30 s), with exercise intensity ≥85% maximum heart rate (HRmax). Based on previous relevant research and considering the specific characteristics of children, HRmax was calculated using the formula: HRmax = 208−0.7 × age (Gelbart et al., 2017). Participants underwent a 6-week formal exercise intervention, consisting of three 24-minute HIIT sessions per week, led by a physical education teacher. The training protocol comprised three parts:

Warm-up (6 min): Exercise intensity maintained at 50%–59% HRmax (heart rate approximately 101-119 bpm). Activities included basic warm-up (*e.g.*, light jogging), dynamic stretching, and neural activation.

Main Session (15 min): The HIIT training program consists of four exercises: 30s jumping jacks, 30s cross jumps, 30s vertical jumps, and 30s burpees. The work-to-rest ratio was 1:1. This circuit was repeated for 3 sets, with 1-minute rest intervals between sets. Total duration was 15 min. Exercise intensity was maintained at ≥85% HRmax (heart rate ≥171 bpm). During rest periods, participants remained stationary and did not engage in any activity.

The exercise content of MICT is 15 min of continuous aerobic running. Including activities like lap running, curve running, and shuttle running. Exercise intensity was maintained at 60%–69% HRmax (heart rate range approximately 121-139 bpm). There were no rest intervals during this phase

Cool-down (3 min): Included stretching and relaxation exercises. Exercise intensity was maintained at 50%–59% HRmax (heart rate approximately 101-119 bpm).

**Figure 1:**
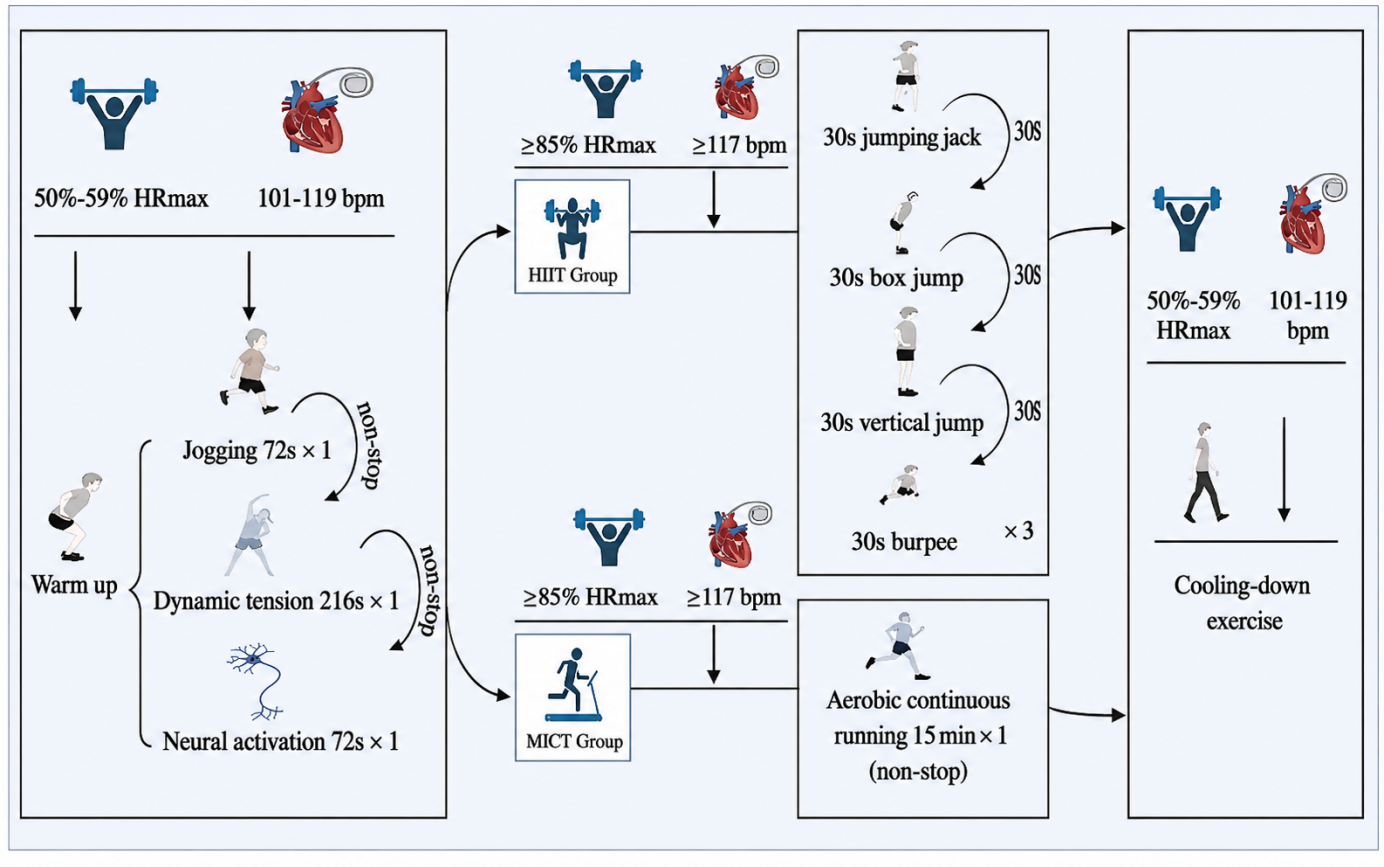
The experimental process diagram.

**Figure 2:**
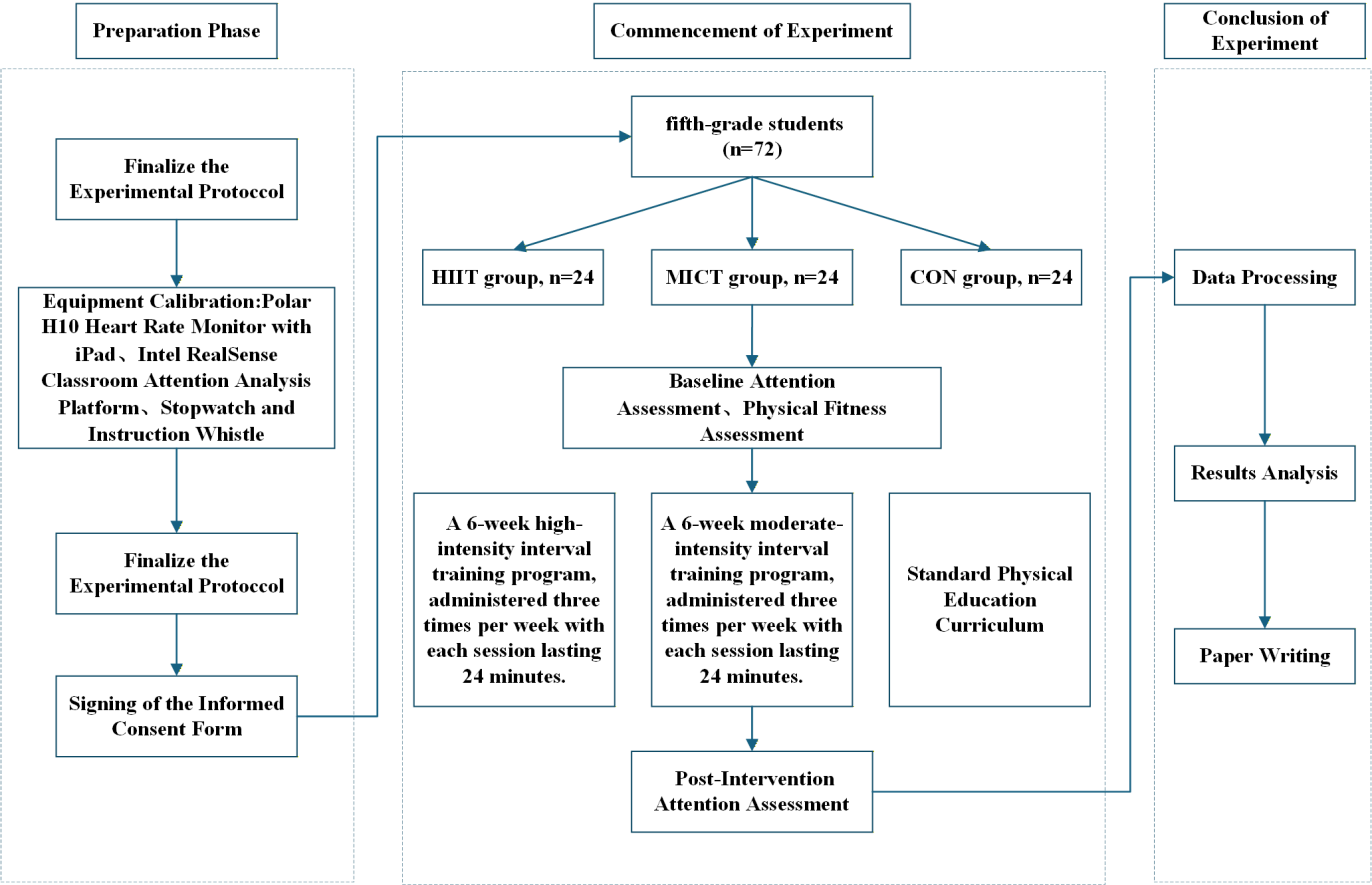
Experimental flowchart.

The detailed exercise intervention protocols for the HIIT and MICT groups are presented in Tables 2 and 3 below. The experimental process diagram and experimental flow chart are shown in Figs. 1 and 2, respectively.

**Table 2:** Summary of the experimental intervention protocol.

Groups	Intervention frequency	Training modality	Work-to-rest ratio	Intervention duration	Exercise intensity
High-intensity interval training group	3 times/week	30 s Jumping Jacks 30 s Lateral Hops 30 s Vertical Jumps 30 s Burpees	1:1	15 min	≥85%HRmax
Moderate-intensity continuous training group		Continuous aerobic running	No intervals		60–69%HRmax

**Table 3:** Single-session training protocol.

Section	Duration	Sets	Exercise	Work-to-rest ratio	Intensity
Warm-up	6 min	1x	Basic Warm-up: Jogging in place(20%) Dynamic stretching(60%) Neuromuscular activation(20%)	No intervals	50–59%HRmax
High-intensity interval training group	15min	3x	30s Jumping Jacks 30s Lateral Hops 30s Vertical Jumps 30s Burpees Interset Rest 1 min	30 s:30 s 1:1	≥85%HRmax
Moderate-intensity continuous training group	15min	1x	Continuous aerobic running	No intervals	60–69%HRmax
Cool-down	3 min	1x	Static stretching Session summary	No intervals	50–59%HRmax

**Experimental quality control:** To ensure the standardization of intervention implementation and the validity of data, the study adopted the following measures: firstly, enhance the cooperation between teachers and parents through pre-communication to promote the compliance of the subjects; all courses are conducted by a uniformly trained teaching team in a fixed and physically matched standardized setting. The possible differences in teaching styles are balanced through a teacher rotation system, while the subjects remain blind to the research objectives. The intensity of exercise is monitored in real time using the Polar heart rate monitoring device, and the training content is dynamically adjusted based on the feedback. Exercise intensity was monitored in real time using a Polar heart rate monitor, which continuously recorded participants’ heart rate data and provided immediate feedback on individual maximum and mean heart rates. Training protocols for both the high-intensity interval training (HIIT) and moderate-intensity continuous training (MICT) groups were subsequently adjusted in accordance with these physiological metrics. The study required the subjects to avoid participating in other systemic physical exercises during the intervention period and to dress uniformly to reduce the interference of additional variables. In addition, by randomly checking course videos and conducting intervention fidelity evaluations, operational consistency is further guaranteed. During the data cleaning stage, the data of subjects who were absent three or more times were excluded to maintain the reliability of the analysis results.

### Attention assessment method

Attention levels were measured before and after the intervention using the Intel RealSense Online Classroom Attention Evaluation System (Ningyu (Shanghai) Technology). The system hardware includes an infrared emitter, dual cameras (infrared and visible light), and an integrated visual processing chip, enabling the capture of depth video streams at a resolution of 1,280 × 720 pixels and a frame rate of 90 fps.

**Figure 3:**
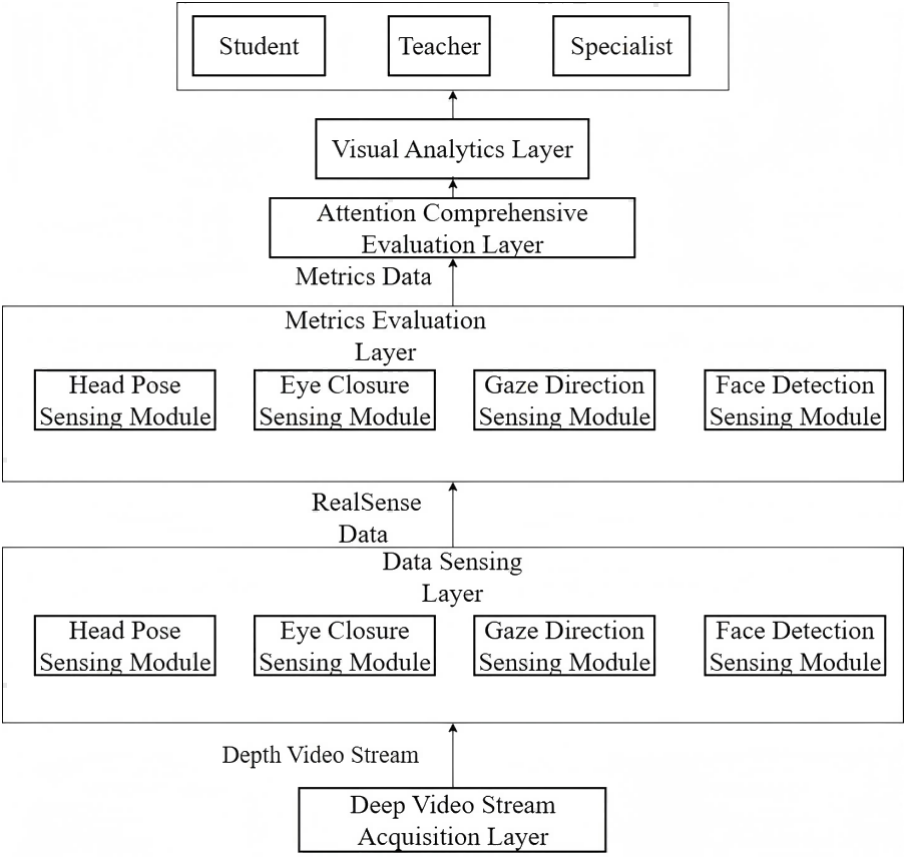
The monitoring results of heart rate under exercise load in the fifth-grade intervention group.

By continuously tracking the three-axis vector of head posture, eye gaze coordinates (determined relative to the classroom screen), eyelid opening and closing states, and facial presence status, the system generates multi-dimensional indicators of students’ attention during classroom activities, The specific testing process is shown in Fig. 3.

The system used in this study is a commercially available classroom attention evaluation tool. All attention indicators were automatically generated by the system’s embedded computer-vision pipeline, and no additional algorithm development, manual feature extraction, or *post hoc* modification of the detection algorithms was performed by the research team.

Raw attention data were screened for completeness prior to analysis. Frames in which facial detection was unsuccessful or system outputs were missing were treated as invalid and excluded from subsequent calculations. Attention indicators for each recording were computed based on valid frames only, using standardized aggregation rules provided by the system.

### Mathematical statistics method

Statistical analyses were performed using SPSS 26.0 software. Statistical analyses followed a predefined and standardized workflow. Raw attention data were first screened for completeness and plausibility, after which valid data were aggregated to obtain sub-indicator scores and composite attention measures. Assumption testing was then conducted prior to inferential analyses to determine the appropriate statistical approach. This stepwise procedure ensured consistency and reproducibility throughout the data analysis process.

Given the relatively small sample size (*n* < 50), the Shapiro–Wilk test was used to assess the normality of data distributions, and Levene’s test was applied to examine the homogeneity of variances. If both assumptions were satisfied, one-way analysis of variance (ANOVA) was used to test baseline homogeneity among the three groups before the intervention and to examine between-group differences after the intervention. In addition, two-way ANOVA was conducted to examine the moderating effect of gender on intervention outcomes.

For longitudinal comparisons, repeated-measures ANOVA was conducted to evaluate the main effects of group, time, and their interaction. When a significant interaction effect was observed (*P* < 0.05), simple effects analyses were performed. If the assumptions for parametric testing were not met, appropriate alternative methods were applied. Welch-corrected ANOVA was used in cases of heterogeneity of variance, and non-parametric tests were applied for non-normally distributed data, including the Mann–Whitney U test or Kruskal–Wallis H test for between-group comparisons and the Wilcoxon signed-rank test for within-group comparisons.

Results were reported as mean ± standard deviation or median, as appropriate. Statistical significance was set at *P* < 0.05 or *P* < 0.01. Partial *η*^2^ effect sizes were reported to facilitate interpretation of intervention effects. Multiple comparisons were adjusted using the Bonferroni correction. Missing data were imputed using the Expectation–Maximization (EM) algorithm, and outliers were identified and handled using box plots.

No manual annotation or rater-based scoring was involved in the attention assessment process; all attention indicators were generated automatically by the system.

Additionally, classroom performance in Chinese, mathematics, and English was synchronously recorded to support the evaluation of intervention effects.

## Results

The primary outcome (in-class attention) was assessed using the Intel RealSense Online Classroom Attention Evaluation System, which automatically generated overall attention scores and four sub-indicators (head posture, gaze direction, eye openness, and facial detection). Heart rate during exercise sessions was monitored using Polar wearable heart rate monitors to verify training intensity.

### Exercise load monitoring

**Figure 4:**
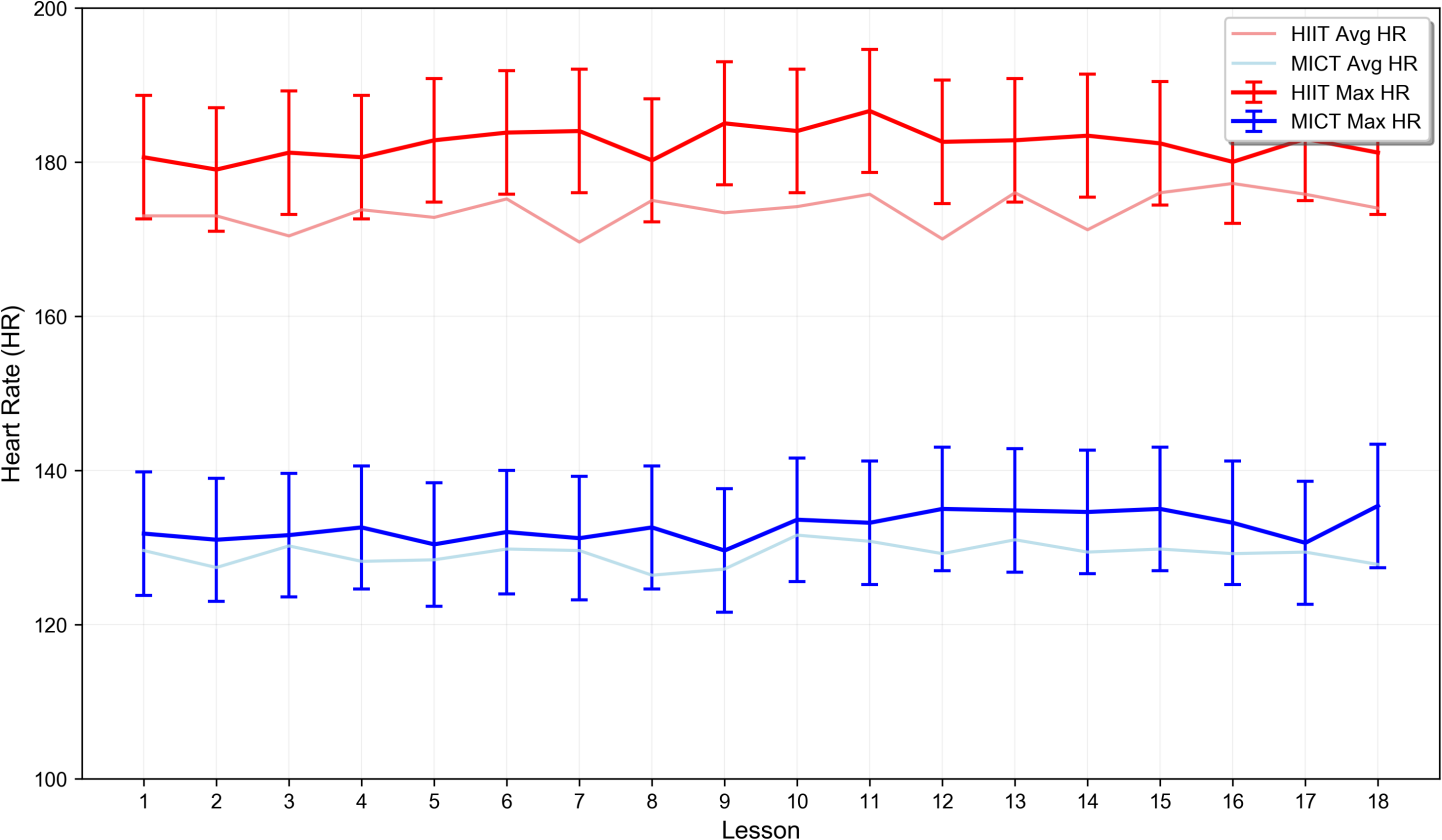
Test data results of the three groups of comprehensive evaluation indicators for children’s attention before and after.

Throughout the three sessions per week on non-consecutive days five children were randomly selected from each training group per session to wear heart rate monitors for exercise intensity monitoring. Average and maximum heart rate data were used to characterize the training load. As shown in Fig. 4, participants in the HIIT group maintained an average heart rate ≥171 beats per minute, corresponding to an exercise intensity of ≥85% HRmax. In contrast, the average heart rate of the MICT group ranged between 121 and 139 beats per minute, corresponding to 60–69% HRmax. These findings indicate that the monitored exercise intensities in both intervention groups were consistent with the predefined target ranges of the study protocol.

### Baseline attention performance

**Overall attention:** Prior to the intervention, no significant differences were observed among the three groups in overall attention scores (*F* = 0.893, *p* = 0.414; Table 4), indicating baseline equivalence.

**Table 4:** Comparison of comprehensive attention metrics at baseline in fifth-grade children prior to intervention.

**Grade**	**CON**	**HIIT**	**MICT**	**ANOVA (F)**	**ANOVA (p)**
Fifth Grade	0.39 ± 0.08	0.38 ± 0.10	0.42 ± 0.13	0.893	0.414

**Notes.**

CON, Control Group; HIIT, High-Intensity Interval Training Group; MICT, Moderate-Intensity Continuous Training Group

**Attention sub-indicators:** Similarly, one-way ANOVA revealed no significant baseline differences among groups for head posture, gaze direction, eye openness, or facial detection (all *p* > 0.05; Table 5), confirming homogeneity across attention-related variables before the intervention.

**Table 5:** Results of the pre-intervention attention assessment at baseline.

Grade	Measures	Groups	M ± SD	ANOVA
Fifth grade	Head pose	HIIT	0.43 ± 0.10	*F*= 1.215 *P* = 0.303
		MICT	0.48 ± 0.09	
		CON	0.45 ± 0.14	
	Gaze Direction	HIIT	0.41 ± 0.09	*F*= 0.557 *P* = 0.576
		MICT	0.42 ± 0.09	
		CON	0.39 ± 0.11	
	Eye Closure	HIIT	0.43 ± 0.13	*F*= 0.106 *P* = 0.900
		MICT	0.44 ± 0.13	
		CON	0.44 ± 0.12	
	Face Detection	HIIT	0.49 ± 0.13	*F*= 0.256 *P* = 0.775
		MICT	0.48 ± 0.10	
		CON	0.50 ± 0.14	

**Notes.**

CON, Control Group; HIIT, High-Intensity Interval Training Group; MICT, Moderate-Intensity Continuous Training Group.

### Post-intervention changes in attention

**Overall attention:** Within-group changes. Repeated-measures ANOVA indicated significant increases in overall attention scores over time in all three groups (*p* < 0.01). Specifically, attention scores increased from 0.39 ± 0.08 to 0.70 ± 0.14 in the HIIT group, from 0.38 ± 0.10 to 0.67 ± 0.12 in the MICT group, and from 0.42 ± 0.13 to 0.55 ± 0.08 in the control group.

**Figure 5:**
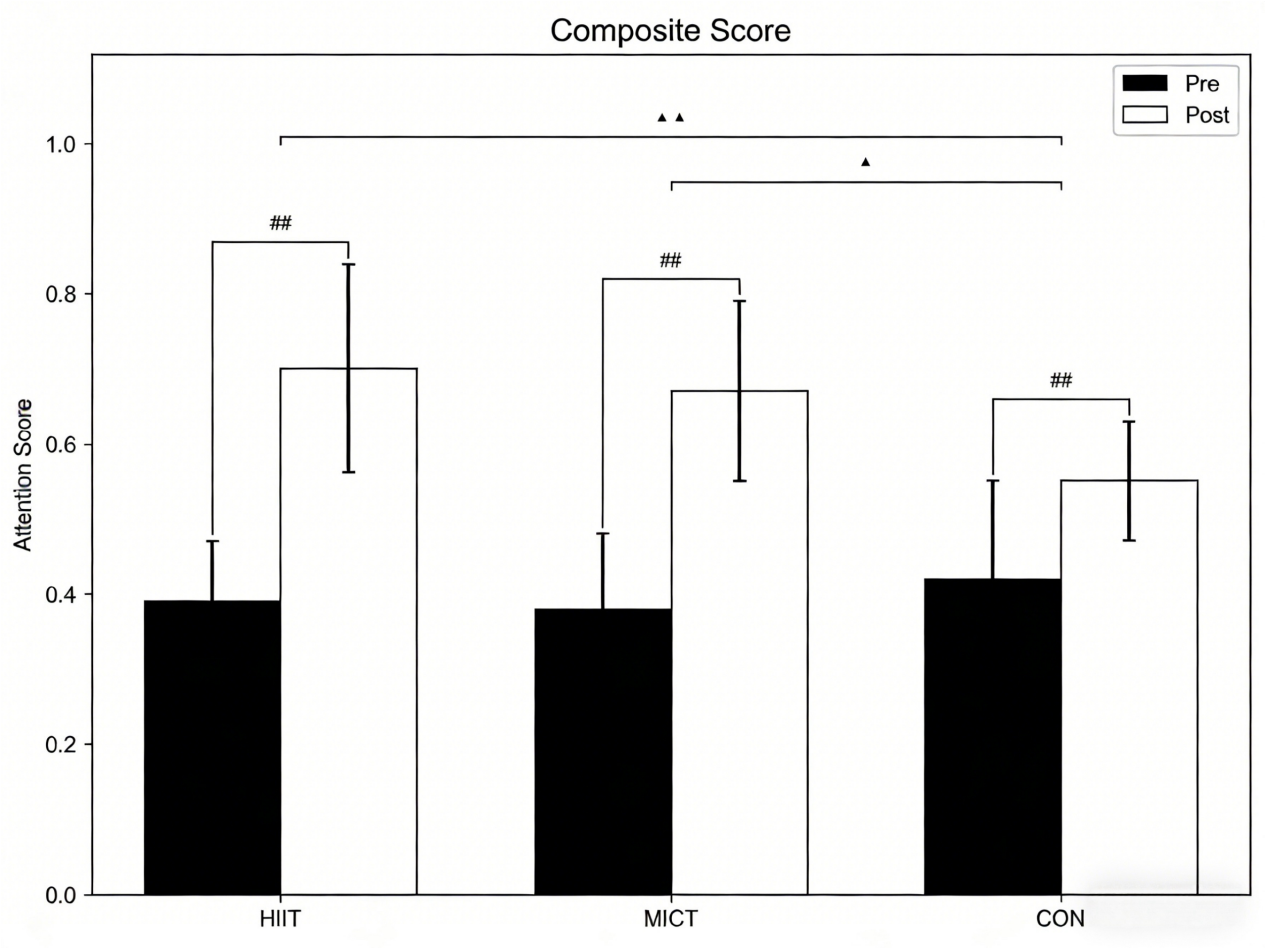
Test data results of the overall attention index among the three groups of children before and after the intervention.

Between-group changes. Analysis revealed a significant main effect of time (*F* = 179.623, *p* < 0.01) and group (*F* = 4.288, *p* < 0.05), as well as a significant time × group interaction (*F* = 9.829, *p* < 0.01). *Post hoc* comparisons showed that post-intervention attention scores in both the HIIT and MICT groups were significantly higher than those in the control group (*p* < 0.01). No significant difference was observed between the two exercise intervention groups (*p* > 0.05). Differences in the magnitude of change across groups are presented in Table 6 and Fig. 5.

**Table 6:** Changes in comprehensive attention measures across three groups of fifth-grade children before and following intervention.

Measure	Groups	Pre-test	Post-test	Time	Time × Group	Group
Comprehensive assessment	HIIT	0.39 ± 0.08	0.70 ± 0.14^##^^▴▴^	179.623^**^	9.829^**^	4.288^*^
	MICT	0.38 ± 0.10	0.67 ± 0.12^##^^▴▴^			
	CON	0.42 ± 0.13	0.55 ± 0.0^##^			

**Notes.**

CON, Control Group; HIIT, High-Intensity Interval Training Group; MICT, Moderate-Intensity Continuous Training Group.

## *P* < 0.01 *vs.* baseline.

▴▴ *P* < 0.01 *vs.* C group.

* Indicates *P* < 0.05, significant difference.

** Indicates *P* < 0.01, highly significant difference.

**Attention sub-indicators:** Within-group changes. Significant pre–post changes were observed in all four attention sub-indicators (head posture, gaze direction, eye openness, facial detection) in both the HIIT and MICT groups (all *p* < 0.01). In the control group, significant changes were observed in gaze direction, eye openness, and facial detection (*p* < 0.01), whereas head posture did not change significantly (*p* > 0.05).

Between-group changes. Two-way repeated-measures ANOVA revealed significant main effects of time and group across all four sub-indicators (all *p* < 0.01). Significant time × group interaction effects were identified for head posture, eye openness, and facial detection (*p* < 0.01), but not for gaze direction (*p* > 0.05).

**Figure 6:**
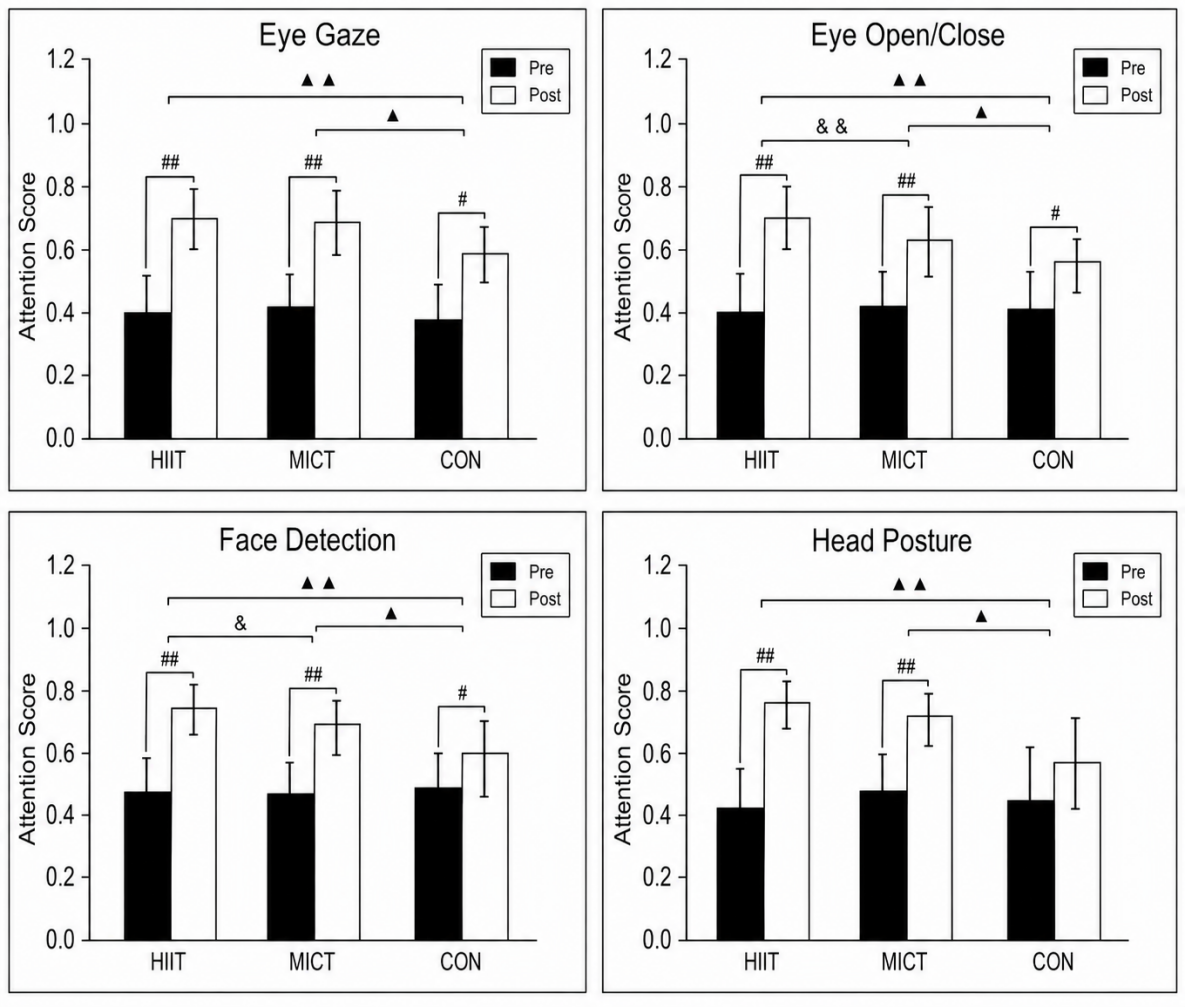
Test data results of the attention sub-indicators among the three groups of children before and after the intervention.

*Post hoc* analyses indicated that both exercise intervention groups exhibited higher post-intervention scores than the control group across the four sub-indicators. Differences between the HIIT and MICT groups varied by indicator, with some sub-indicators showing significant between-group differences and others showing no significant differences. Changes in sub-indicator scores and their magnitudes are detailed in Table 7 and Fig. 6.

**Table 7:** Changes in component attention measures across three groups of fifth-grade children before and following intervention.

Measures	Groups	Pre-test	Post-test	Time	Time × Group	Groups
Head Pose	HIIT	0.43 ± 0.10	0.80 ± 0.11^##^^▴▴^	161.812^**^	14.225^**^	14.624^**^
	MICT	0.48 ± 0.09	0.75 ± 0.11^##^^▴▴^			
	CON	0.45 ± 0.14	0.56 ± 0.14			
Gaze Direction	HIIT	0.41 ± 0.09	0.71 ± 0.08^##^^▴▴^	245.568^**^	2.890	7.261^**^
	MICT	0.42 ± 0.09	0.70 ± 0.13^##^^▴▴^			
	CON	0.39 ± 0.11	0.59 ± 0.09^#^			
Eye Closure	HIIT	0.43 ± 0.13	0.78 ± 0.11^##^^&&^^▴▴^	136.371^**^	10.670^**^	7.883^**^
	MICT	0.44 ± 0.13	0.65 ± 0.12^##^^▴^			
	CON	0.44 ± 0.12	0.57 ± 0.10^#^			
Face Detection	HIIT	0.49 ± 0.13	0.78 ± 0.10^##^^&^^▴▴^	112.054^**^	8.451^**^	5.240^**^
	MICT	0.48 ± 0.10	0.72 ± 0.11^##^^▴▴^			
	CON	0.50 ± 0.14	0.61 ± 0.14^#^			

**Notes.**

CON, Control Group; HIIT, High-Intensity Interval Training Group; MICT, Moderate-Intensity Continuous Training Group.

# *P* < 0.05 *vs.* baseline.

## *P* < 0.01 *vs.* baseline.

& *P* < 0.05 *vs.* MICT group.

&& *P* < 0.01 *vs.* MICT group.

▴ *P* < 0.05 *vs.* C group.

▴▴ *P* < 0.01 *vs.* C group.

** Indicates *P* < 0.01, highly significant difference.

## Discussion

In this study, we compared the effects of 6-week high-intensity interval training (HIIT) and moderate-intensity continuous training (MICT) on the attention of fifth-grade children. The results showed that children’s attention improved regardless of the training modality. Notably, the improvement amplitude of the comprehensive attention index in fifth-grade children after HIIT training (79.5%) was significantly higher than that in the MICT group (76.3%) (Lubans et al., 2016), and HIIT showed superior promotional effects on attention sub-dimensions such as head posture and eye openness/closure (Yin, Zhou & Lan, 2020). This finding is particularly important for elementary school-aged children, as this period represents a critical stage for attention and cognitive function development, and improved attention is considered an important prerequisite for learning-related behaviors and classroom engagement, which may indirectly support academic performance, social skills, and physical–mental health. HIIT is a training mode that alternates between high-intensity exercise and low-intensity recovery, providing efficient training effects within a short time. Existing studies have shown that HIIT can promote attention development by enhancing neuroplasticity and cerebral cortical activation. It should be noted that the mechanisms discussed below, while supported by prior literature, are proposed as plausible explanations for our observed effects and would benefit from direct measurement in future studies.First, HIIT may enhance neuroplasticity in children’s brains. During the developmental stage, children’s brains exhibit strong synaptic plasticity and high adaptability to external stimuli. High-intensity exercise can accelerate the secretion of brain-derived neurotrophic factor (BDNF), which plays a key role in synaptic plasticity, learning, and memory. Studies have shown that HIIT can increase BDNF expression, thereby potentially enhancing neuroplasticity and improving cognitive function (Hu et al., 2021). Second, HIIT may increase neuronal activation levels (Buzdagli et al., 2024). Short-term high-intensity exercise is proposed to increase cerebral blood flow and oxygen supply, thereby promoting brain metabolic activity and improving cognitive functions such as attention. The peak intensity of HIIT may stimulate the prefrontal cortex, leading to better performance in post-exercise cognitive tasks. For fifth-grade children, this short-duration yet efficient exercise mode may rapidly activate brain cognitive regions, thereby enhancing their concentration in class. Furthermore, from a psychological perspective, HIIT may improve children’s arousal level and executive function (Andrade-Lara et al., 2023) through factors such as greater task novelty, frequent opportunities for immediate feedback, and enhanced perceived self-efficacy, which could support sustained attentional engagement. Short-term high-intensity exercise can enhance sympathetic nervous system activity, enabling individuals to maintain a high level of attention concentration in the short term after exercise. In contrast, although MICT has positive effects on overall health, its role in improving short-term attention is relatively weaker.

MICT typically involves prolonged moderate-intensity exercise, such as continuous aerobic running. Studies have shown that MICT can promote overall brain health by improving cardiopulmonary function and enhancing aerobic metabolism (Mahar et al., 2006). Compared with HIIT, MICT has lower intensity but longer duration, so its improvement in cognitive function tends to be more gradual and stable over time (Vanhelst et al., 2016). The mechanisms underlying MICT’s effects on attention improvement may mainly involve three aspects: First, MICT may enhance prefrontal cortex function. Through long-term aerobic exercise, MICT could increase blood supply to the prefrontal cortex, improving executive function and self-regulation ability (Liu et al., 2023). For fifth-grade children, sustained moderate-intensity exercise helps develop attention stability and task persistence. Second, MICT may promote neurotransmitter regulation. Moderate-intensity exercise might help stabilize dopamine and norepinephrine levels, which play crucial roles in attention regulation. MICT could increase the baseline levels of these neurotransmitters through long-term exercise, making it easier for children to maintain attention in daily learning environments (Gelabert et al., 2023). Additionally, MICT may improve brain oxygen supply efficiency, thereby enhancing attention stability and cognitive function in the long term (Hillman et al., 2009). However, compared with HIIT, the short-term improvement effect of MICT on attention may be less significant (Wu et al., 2023). Studies have shown that MICT often requires a longer duration to produce observable effects on cognitive function, whereas HIIT can bring more immediate attention improvement in a shorter time (Pereira, Lino & Andrade-Moraes, 2023). This may partly explain why the attention improvement amplitude of fifth-grade children in this study was higher after HIIT training (79.5%) than after MICT (76.3%). Furthermore, this result may be related to the characteristics of children’s cognitive development. Fifth-grade children (10–11 years old) have a maturing attention system with high plasticity, so they may benefit from different types of exercise training and exhibit strong physiological adaptability to high-intensity stimuli, leading to superior performance in HIIT intervention.

### The impact on the sub-indicators of attention

The present study further analyzed four sub-indicators of attention—head posture, gaze direction, eye openness, and facial detection—using intelligent video capture technology. Both HIIT and MICT significantly improved all four measures compared with the control group, with HIIT showing the greatest overall improvements. Exercise involving rapid directional changes and dynamic balance (*e.g.*, jumping jacks, burpees) stimulates the vestibular system and proprioceptive feedback, potentially enhancing postural control and gaze stability (Elliott et al., 2024). Strengthening of core musculature may also contribute to improved head posture, which in turn supports steady visual tracking during classroom learning. The HIIT group required rapid action switches within set times, while the MICT group needed to adjust their running paths as required. These tasks may engage neural systems involved in spatial attention allocation and visual information tracking, enhancing an individual’s focus and stability on visual targets. The eye opening/closing state may be related to mental state. Exercise can significantly elevate mood, reduce feelings of fatigue, and this positive emotional state may be reflected in eye movement characteristics (Thom et al., 2021). The eyes are core to observing the external world; the degree of eye openness is related to the observable eye area. Greater focus is often associated with a larger exposed eye area. Furthermore, face recognition and eye recognition are closely linked; face recognition is a prerequisite for eye recognition, which in turn is a prerequisite for assessing student concentration in class. Facial expressions are external manifestations of an individual’s emotional state. Exercise increases the secretion of endorphins and dopamine, enhancing feelings of pleasure. For instance, HIIT can increase sympathetic nervous system excitement, potentially improving emotional expression (Jung, Bourne & Little, 2014), while MICT may help improve emotion regulation, making individuals more likely to display positive facial expressions. This positive state may enhance the efficiency of cognitive resource allocation, leading to more stable and focused attention, which is then reflected in the facial detection metrics.

Based on the pre-test results of this study, the three groups of fifth-grade children showed no significant differences in the four attention sub-indicators. After the 6-week intervention, the performance of the fifth-grade children in class was as follows: the improvement magnitudes for all four sub-indicators were higher in the HIIT and MICT groups compared to the control group, and the HIIT group outperformed the MICT group. The order of improvement magnitude for the sub-indicators was: HIIT group: Gaze Direction (38.1%) > Head Posture (37.0%) > Eye Opening/Closing (35.0%) > Facial Detection (29.0%); MICT group: Gaze Direction (28.0%) > Head Posture (27.0%) > Facial Detection (24.0%) > Eye Opening/Closing (21.0%). Therefore, both 6-week HIIT and MICT interventions can significantly improve the attention of fifth-grade children, but the degree of improvement varies across different indicators. This may be related to factors such as the match between exercise mode and cognitive demands, exercise intensity, neurophysiological mechanisms, and the children’s age and cognitive development stage (Bulten et al., 2022). The significant advantage of the HIIT group in the Gaze Direction indicator (38.1% improvement) in this study might be related to the acute activation of the prefrontal-parietal network. High-intensity exercise can rapidly increase blood oxygen levels in the dorsal anterior cingulate cortex, a region implicated in prioritizing visual targets. The enhancement of its function may optimize the precision of eye movement control (Verret et al., 2012). In contrast, head posture involves vestibular-proprioceptive integration and may require a longer training period to achieve neural adaptation. The prominent performance of the MICT group in the Gaze Direction indicator might be associated with the improvement of long-term visual tracking ability. Moderate-intensity exercise potentially upregulates serotonin (5-HT) synthesis (Lin & Kuo, 2013) and reduces the sensitivity of the amygdala to negative emotions, thereby promoting stable visual focus. This emotion–cognition synergy might enhance visual attention during classroom interactions through the mirror neuron system (MNS) (Oberman et al., 2005), whereas improvement in the Eye Opening/Closing state might be slower due to insufficient suppression of the default mode network.

### The impact on the comprehensive index of attention

Under the same intervention format, the improvement magnitudes of the various sub-indicators differed. This may be related to the distinct physiological and psychological mechanisms triggered by exercise. The interaction of these mechanisms collectively constructs the comprehensive effect of attention enhancement. The improvement effects of different intervention formats may vary due to differences in exercise intensity, duration, individual differences, and the degree of activation of different brain regions and neuroplasticity induced by the type of exercise.

Based on the pre-test results, no significant differences were observed among the three groups of fifth-grade children in the comprehensive attention indicator. After the 6-week intervention, the magnitude of improvement in comprehensive attention was greatest in the HIIT group, followed by the MICT group, and was lowest in the control group.Therefore, both HIIT and MICT can effectively enhance the comprehensive attention level of fifth-grade children, with HIIT showing a better improvement effect.

The results of this study show that after the 6-week intervention, both exercise intervention formats had varying degrees of promotive effects on the overall attention level of fifth-grade children, which is consistent with previous research. The superior improvement effect of the HIIT group might be attributed to multiple factors. On one hand, the intermittent challenges of HIIT (*e.g.*, movement switches, time control) may dynamically match children’s motor abilities, potentially inducing high immersion and thereby increasing cognitive engagement. Simultaneously, the diverse movement design of HIIT (*e.g.*, cross jumps, burpees) may offer greater novelty and engagement than continuous steady-state exercise. Through these novelty stimuli, it may promote dopamine release, enhance exercise pleasure, stimulate students’ interest in movement, and motivate children to actively increase their attention to learn these movements, consequently improving student attention (Jones et al., 2021). Children are more likely to receive immediate feedback from achieving phased goals (*e.g.*, completing a set of movements) in HIIT, potentially enhancing self-efficacy, which then may positively transfer to the maintenance of classroom attention (Jiang, Wang & Qin, 2025). In contrast, the steady-state nature of MICT might lead students’ attention to shift from movement control to environmental monitoring (*e.g.*, route adjustment), potentially redistributing some cognitive resources. On the other hand, the higher exercise intensity of HIIT may induce stronger acute neurophysiological responses. HIIT may increase lactate release, activating monocarboxylate transporters and promoting the transmembrane transport of lactate as a neuronal energy substrate, thereby potentially enhancing prefrontal metabolic efficiency (De Castro Abrantes, Briquet & Schmuziger, 2019). Concurrently, by rapidly elevating blood levels of brain-derived neurotrophic factor (BDNF) and norepinephrine, it may preferentially enhance the functional connectivity between the prefrontal cortex and the striatum, optimizing working memory and inhibitory control, thus leading to superior performance on the comprehensive attention indicator (Chaddock-Heyman et al., 2018). MICT may improve sustained attention and interference control by promoting processes such as hippocampal neurogenesis, but its effect on the comprehensive indicator might be more gradual, and the manifestation of higher cognitive benefits may require longer-term intervention (Marcourt et al., 2025).

### Study strengths and practical implications

This study possesses several strengths. It employed a randomized controlled design conducted within the authentic setting of regular school physical education classes, enhancing the ecological validity of the findings. The use of an objective, intelligent video-based assessment system for attention measurement reduces subjective bias and allows for the analysis of specific behavioral sub-components. Furthermore, exercise intensity was rigorously monitored and controlled using heart rate monitoring, ensuring intervention fidelity. From a practical perspective, our findings provide empirical support for integrating HIIT as a time-efficient module into primary school physical education curricula. Given that HIIT demonstrated superior short-term benefits on attention in a relatively brief intervention period, it represents a viable strategy for schools facing curricular time constraints, offering a means to enhance students’ cognitive readiness for learning alongside physical fitness.

## Conclusions

**Conclusions:** This study examined the effects of high-intensity interval training (HIIT) and moderate-intensity continuous training (MICT) on classroom attention in fifth-grade children. The results indicate that both HIIT and MICT significantly improved overall attention compared with the control condition, with HIIT demonstrating a greater enhancement effect. Although children in the control group who maintained regular physical activity also showed improvements in attention, these gains were smaller than those observed in the two structured exercise intervention groups.

Further analysis of attention sub-indicators revealed distinct response patterns across exercise modalities. Following HIIT intervention, improvements were greatest in gaze direction, followed by head posture, eye openness/closure, and facial detection. In the MICT group, the order of improvement was gaze direction, head posture, facial detection, and eye openness/closure. These findings suggest that while both exercise formats effectively enhance attention, HIIT may provide superior short-term benefits, particularly for visual attention and postural-related indicators.

**Limitations:** (1) The sample size was limited to 72 participants, all recruited from a single regional school, resulting in insufficient sample representativeness. This may restrict the generalizability of the results to children in different geographical regions or with diverse demographic characteristics.

(2) The 6-week intervention duration was relatively short, failing to fully reflect the persistence of exercise-induced attention improvements. The absence of long-term intervention and follow-up studies may compromise the temporal validity of the conclusions.

(3) Attention assessment relied solely on intelligent video capture technology, lacking cross-validation with objective data from neurophysiological indicators, leading to a single-dimensional evaluation approach.

**Future directions:** (1) Future research could expand the sample size to include different school stages (*e.g.*, all elementary grades) and multi-regional schools, incorporating children from urban and rural areas with diverse physical fitness characteristics to enhance the representativeness and generalizability of the results.

(2) Extend the intervention duration to 12 weeks or longer, with the addition of 6–12 months of follow-up observations, to systematically investigate the long-term impact and maintenance effects of exercise on children’s attention.

(3) Integrate physiological indicators such as brain function imaging (*e.g.*, fMRI) and neurotransmitter levels (*e.g.*, BDNF) to deeply explore the neural mechanisms underlying exercise-induced attention improvements, thereby enhancing the scientific rigor of the research.

## Supplemental Information

10.7717/peerj.21580/supp-1 Supplemental Information 1 Original data

## Additional Information and Declarations

### Data Availability

The following information was supplied regarding data availability: The raw data is available in the Supplemental File.
